# Developing and implementing a service charter for an integrated regional stroke service: an exploratory case study

**DOI:** 10.1186/1472-6963-14-141

**Published:** 2014-03-29

**Authors:** Jean-Pierre Thomassen, Kees Ahaus, Steven Van de Walle

**Affiliations:** 1 University of Groningen, Mathenesserlaan 330, 3021 HZ, Rotterdam, The Netherlands; 2 Faculty of Economics and Business (Department of Operations), University of Groningen, Nettelbosje 2, 9747 AE, Groningen, The Netherlands; 3 Department of Public Administration, Erasmus University Rotterdam, Burgemeester Oudlaan 50, 3062 PA, Rotterdam, The Netherlands

**Keywords:** Service charter, Enablers, Integrated care, Implementation, Patient-centeredness

## Abstract

**Background:**

Based on practices in commercial organizations and public services, healthcare organizations are using service charters to inform patients about the quality of service they can expect and to increase patient-centeredness. In the Netherlands, an integrated regional stroke service involving five organizations has developed and implemented a single service charter. The purpose of this study is to determine the organizational enablers for the effective development and implementation of this service charter.

**Methods:**

We have conducted an exploratory qualitative study using Grounded Theory to determine the organizational enablers of charter development and implementation. Individual semi-structured interviews were held with all members of the steering committee and the taskforce responsible for the service charter. In these twelve interviews, participants were retrospectively asked for their opinions of the enablers. Interview transcripts have been analysed using Glaser’s approach of substantive coding consisting of open and selective coding in order to develop a framework of these enablers. A tabula rasa approach was used without any preconceived frameworks used in the coding process.

**Results:**

We have determined seven categories of enablers formed of a total of 27 properties. The categories address a broad spectrum of enablers dealing with the basic foundations for cooperation, the way to manage the project’s organization and the way to implement the service charter. In addition to the enablers within each individual organization, enablers that reflect the whole chain seem to be important for the effective development and implementation of this service charter. Strategic alignment of goals within the chain, trust between organizations, willingness to cooperate and the extent of process integration are all important properties.

**Conclusions:**

This first exploratory study into the enablers of the effective development and implementation was based on a single case study in the Netherlands. This is the only integrated care chain using a single service charter that we could find. Nevertheless, the results of our explorative study provide an initial framework for the development and implementation of service charters in integrated care settings. This research contributes to the literature on service charters, on patient-centeredness in integrated care and on the implementation of innovations.

## Background

Strokes are a common cause of premature death and disability and present a major global public health challenge [1]. They are one of the leading causes of death in the Netherlands [2]. Based on the concept of integrated care, regional stroke services have been established in the Netherlands. Integrated care is an organizational coordination process that seeks to achieve seamless and continuous care that is tailored to the patient’s needs and based on a holistic view of the patient [3]. Three phases of the integrated stroke service can be distinguished: acute care involving the emergency department and stroke unit of the regional hospital, rehabilitation involving rehabilitation centres, specialized nursing homes and home care and, finally, long-term support. Delivering optimal care with this range of providers requires a complex mix of collaboration on operational and individual levels involving streamlining information flows and the transfer of acute patients. On a tactical level, this can involve performance indicators on the care-chain level and, on the strategic level, financial and logistical agreements [4]. These interventions aim to improve patient care and medical outcomes, objectives that fit into the general goals of care integration: enhancing patient satisfaction and quality of life, efficiency and outcomes [5].

A service charter is a statement laying out the service level that patients can expect and what the organization will do if it fails to deliver [6]. Normally, it consists of four design elements one or more promises to patients; the tangible or intangible compensation in the event of service failure; the process to invoke the charter; and other charter characteristics. In our setting, a charter should transform an intangible health product into a measurable expectation for the patient prior to receiving the healthcare service. The concept of the service charter was originally developed in commercial organizations [7] and was then adopted by public services and healthcare. In the United Kingdom, the concept has been used in all NHS hospitals since 1991 in the form of the Patient’s Charter [8]. Healthcare organizations in Italy [9], the USA [10] and the Netherlands [11] have also adopted the concept. In several Dutch healthcare services, the multi-attribute specific service charter [12] is used. This consists of a number of promises covering the patient’s journey from general practitioner referral through to discharge from the hospital and follow-up arrangements. The specific goals in implementing service charters are: to increase the responsiveness of healthcare services to the wishes of patients; to make healthcare services more accountable; to ensure patients know what to expect so that they can become more equal partners in the healthcare process; to be used as a listening mechanism; to increase feedback from patients; and to improve patient satisfaction. Further, communicating a service charter is seen as a signal of patient-centeredness that could have beneficial marketing effects [13]. Following the use of service charters in commercial organizations and public services, healthcare organizations have started using the concept in order to improve patient satisfaction by improving their patient-centeredness.

Based on their extensive research of the literature on service charters, Hogreve and Gremler [14] have concluded that it is still challenging for scholars to show whether, and how, service charters are helpful in increasing service quality. The literature on the enablers of service charter implementation is dominated by citations and anecdotal evidence. Hays and Hill [15] have conducted intensive empirical research on the effects of using service charters on employees and the service development process. Thomassen et al. [16] have conducted a concept mapping study involving an integrated Delphi study on the enablers for implementing service charters within individual public sector organizations. However this article contributes by offering empirical research on operational aspects of service charter development and implementation in an integrated care setting. An extensive healthcare and management literature review has resulted in the conclusion that no empirical research has been published before on the implementation of a single service charter in a chain.

Our research focusses on both the development and implementation of a service charter. Implementation is the process of putting the service charter into use, making the service charter work in daily practice and institutionalizing it [17]. Implementing a service charter often requires a new set of behaviours, routines and ways of working that are directed at improving patients’ experiences. In our research, we focus on enablers. Enablers are those elements of processes, structures or states that are necessary antecedents for the successful development and implementation of a service charter [18]. Depending on their qualities, they can hamper or promote the effective development and implementation of a service charter [19]. Within the regional stroke service, the term effectiveness refers to the aggregated consistency, quality and appropriateness of service charter development and use [20] in achieving the two goals of the service charter: improving the patient-centeredness of the chain and increasing patient satisfaction.

It has been estimated that two-thirds of all innovation implementations in healthcare fail [21] and there is evidence of failures in the development and implementation of service charters. The literature presents several cases where service charter implementation has failed due to the absence of necessary enablers. Examples include a lack of involvement by employees and middle managers leading to an inconsequential use of service charters in daily practice [22] and the service charter becoming seen as a disciplinary device or as a criticism of the service offered [23] resulting in the charter being targeted for serious criticisms by employees [24]. Ohemeng [25] concludes that staff resistance to customer-oriented change is one of the most underestimated aspects of introducing a service charter.

Our research contributes to the literature on the development and implementation of service charters and on the patient-centeredness of integrated care. It is the first piece of research to focus on a single service charter for a chain of health organizations. The research question driving our exploratory research is: *what, according to experienced practitioners in an integrated regional stroke service, are the important enablers of an effective development and implementation of a single service charter for the chain?*

We now describe the setting of our case study. This is followed by a description of the method used consisting of in-depth interviews and analysis based on Grounded Theory. Following this, we present the results of our research followed by a discussion and conclusions.

### Setting

Integrated stroke service in the Netherlands can be distinguished in three phases. Phase 1 consists of acute care involving the emergency department and stroke unit of the regional hospital. Phase 2 consists of rehabilitation involving rehabilitation centres, specialized nursing homes and home care and finally phase 3 consists of long-term support. The regional stroke service in this research is located in the Southern part of the Netherlands and is defined by five participating organizations involved in the first two phases. The services for patients are delivered by a regional hospital, a rehabilitation centre and three specialized nursing homes that offer similar services but are located in various places across the region. Patients tend to be frail elderly people who have had a stroke. On the strategic level, a covenant for intensive cooperation has been signed by the boards of the five organizations. A tactical-level steering committee with managers from the five involved organizations develops and implements strategies that aim to optimize processes and improve patient satisfaction and reduce costs. This steering committee has been the initiator behind implementing the service charter for the stroke service.

The formal goals of the regional stroke service in developing and implementing a service charter were to improve the patient-centeredness of the process and to increase patient satisfaction. After the decision by the steering committee to use a service charter, a project organization was set up. The existing steering committee was in charge of the whole project, and a taskforce on the chain level with representatives of all the organizations was responsible for implementation activities. In a later stage, additional working groups were established at each organization. A project leader working for the hospital was put in charge of overall project management. After consensus was reached on starting the project, it was decided in May 2009 that there should be a single overall service charter for the whole chain and additional sub-charters for the hospital and the various possible patient flows on leaving the hospital. This was in order to avoid overloading patients with information. An approach consisting of three stages has been used to realize the objectives of the project: developing the service charter; taking measures to realize the content; and finally developing measures to sustain and continuously improve the charter.

In the development of the service charter, research was conducted by the organizations among recent stroke patients in order to gain a clear picture of patient satisfaction and patients’ expectations and preferences. A survey provided an overall view of patient satisfaction with the various stages in their journey through the system. After the survey the subsequent two focus groups with patients and an additional thirty individual in-depth interviews, provided insights into patients’ expectations and preferences. Further, employees were consulted on what they thought was important for patients. Based on the results of this process, the steering committee and taskforce developed a first draft of a service charter that consisted of a set of overall promises for the whole chain (see Table 1) and more specific sets of promises for the various flows after leaving the hospital.

**Table 1:** Promises in the overall service charter

**The integrated stroke service is a collaboration of five organizations**	
1	All relevant information concerning you will be present on transfer to the next partner(s) involved in your care.
2	The stroke-service partners will conduct an intake interview. This will provide you and/or your family/carer with information about the organization and the department. You will receive general information on paper.
3	You and/or your family/carer will be welcomed by our staff in a hospitable way and treated with respect.
4	You and/or your family/carer will be intensively involved in all decisions made during the medical treatment and rehabilitation process. The treatment will be fine-tuned to be as close as possible to your situation and wishes.
5	A medical treatment plan will be drawn up. This will be discussed by the doctor with you and/or your family/carer.
6	Your questions will be answered within 24 hours.

The development of the service charter involved intensive co-operation between the steering committee, the taskforce and the working groups in the five organizations. This was intended to generate commitment to the content of the service charter and to ensure that it could be realized. Given that the steering committee had aimed to develop service promises that could easily be realized by all the involved organizations, the improvement measures required for realizing the content were perceived by the organizations as feasible. By the end of 2010, the internal and external communication campaigns had started. In several organizations, seminars were organized to present the service charter. Following this, although the project organization was still in place, the approach switched from a collective approach to one based on the individual organizations. Thus, in the subsequent phases of taking measures to realize the content of the service charter and then sustaining it, there was less central coordination.

## Methods

In our research, we used a case-study approach since case studies have proven their usefulness in the development of new theories [26]. We had the opportunity to carry out the research while the implementation process was ongoing, albeit approaching its end. The authors’ role was that of researcher, and they were not involved in the actual development or implementation. The case is unique in that no further cases are known in practice or in the literature where a chain of organizations has developed and implemented a service charter. Since this case is distinct from other implementations, there was no other option but to use a single case study approach. The uniqueness is the rationale for this single-case design [27].

In our research, we drew on the opinions of experienced practitioners who were responsible for the development and implementation of the service charter. We decided not to work with a sample but to involve all the members of the steering committee representing the five organizations. These members are managers working for the five organizations involved. Further, the current five members of the service charter taskforce, the chain coordinator and the head of the supporting hospital department that coordinated the patient research were interviewed. All practitioners we have invited to participate have done so. We have thus involved all the people responsible for and intensively participating in the development and implementation of the service charter in the chain and in its individual organizations. We obtained informed consent in writing from the participants to use and publish quotes as stated in their interviews on an anonymous basis. For this research no approval of a medical ethical committee was required according to the criteria of the Dutch law ‘Medical-scientific research among people (WMO)’ of December 1^st^ 1999.

**Figure 1:**
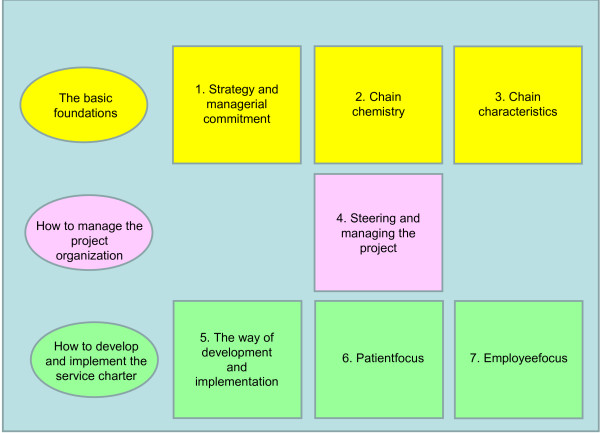
The seven categories of enablers.

Given that no frameworks exist for the enablers of an effective development and implementation of one single service charter for a chain, we used Grounded Theory to develop theory based on our data. This is an established exploratory qualitative method for analysing empirical data in order to build a general theory. After acquiring the commitment of the steering committee to start the research, relevant documents such as project documents and the results of the research among patients were obtained and studied. Individual interviews were held, and the respondents were assured of anonymity to encourage them to speak freely and not be influenced by others. Interviews are seen as a highly efficient way to gather rich empirical data, especially when, as in our situation, the phenomenon of interest is highly episodic and infrequent [28]. A total of twelve semi-structured interviews, each of approximately 1.5 hours were conducted by a single trained interviewer. An interview protocol was used (see Table 2) with open questions, seeking explanations of how the service charter was developed and implemented, the perceived enablers on both the organizational and chain levels, and what went well and what was problematic. Participants were encouraged to speak freely and share their opinions, successes and failures during the development and implementation. All the interviews were taped and later transcribed in detail in order to capture the full richness of the respondents’ views. For transcript analysis we used Glaser’s ‘tabula rasa approach’ for coding [29] and avoided forced coding based on preconceived frameworks. We used a substantive coding approach consisting of open, followed by selective, coding [30]. For the open coding, the interview transcripts were reviewed line-by-line by three independent researchers, and codes were placed in the margins of the transcripts. In an extensive consensus meeting, the three researchers established an agreed set of 44 codes. Following this, all the text fragments from the twelve transcripts were reorganized based on these 44 codes. To obtain an overview for each code, all the fragments linked to a code were combined in a single document. For each code, several sub-codes were then determined. Finally, the codes and sub-codes were analysed and re-analysed for emerging patterns and themes. This selective coding finally resulted in seven categories with a total of 27 properties (see Figure 1). The seven categories can be divided into three overarching themes: ‘the basic foundations’, ‘how to manage the project organization’ and ‘how to develop and implement the service charter’.

**Table 2:** Interview protocol

		**5 steering committee members and 5 taskforce members (10 interviews)**	**Chain coordinator (1 interview)**	**Support on patient research (1 interview)**
**A. The organization**				
1	Which part(s) of the organization are involved in the service charter	x		
2	How many employees are working here?	x		
**B. Development and implementation of service charter**				
1	How was the service charter developed on the chain level? What was the process?	x	x	x
2	How has the service charter been developed and implemented in your organization?	x		
3	What were the goals in implementing a service charter?	x	x	x
**C. Enablers for the effective implementation in your organization. If you look back…**				
1	What went well during the implementation in your organization?	x		
2	What would you have done differently?	x		
3	What were the most important enablers of effective implementation in your organization?	x		
4	Imagine that another organization wanting to implement a service charter asks for your advice, what must it have in place in order to implement it effectively?	x		
**D. Enablers for the effective implementation for the whole chain. If you look back…**				
1	What went well during the implementation in the whole chain?	x	x	x
2	What would you have done differently?	x	x	x
3	What are the most important enablers of effective implementation on the chain level?	x	x	x
4	Imagine that another organization wanting to implement a service charter asks for your advice, what must it have in place in order to implement it effectively?	x	x	x

This is the interview protocol used for the twelve interviews. The last three columns indicate (with an X) which questions were asked to which categories of interviewee.

## Results

The extensive analysis based on Grounded Theory has resulted in seven categories of enablers. These categories are derived from the empirical study without using any predetermined or existing model or framework. The categories and their 27 associated properties are presented in Table 3.

**Table 3:** Enablers: categories and properties

**1. Strategy and managerial commitment**	
*A.*	*Strategic alignment*: The service charter fits the vision of all involved organizations and the goals of the chain.
*B.*	*A concrete and shared goal*: There is a clear goal in using the service charter that is genuinely supported by all the organizations involved. It is to improve patient-centeredness and customer satisfaction.
*C.*	*Commitment and support*: All the organizational boards fully understand the concept of a service charter, the consequences of using it and are committed to using it.
*D.*	*Resources*: The involved organizations dedicate the appropriate people and other resources to the project.
**2. Chain chemistry**	
*A.*	*Trust*: Being open and transparent with each other and trusting in the other organizations that the initiative will be successful.
*B.*	*Willingness to cooperate*: The commitment of all organizations to develop a single service charter for the whole chain. The capacity to listen to and to respect the opinions of the other organizations involved in the chain.
*C.*	*Chain before organization*: Having the courage to prioritize the chain over the interests of the individual organization.
*D.*	*One organization in the lead*: Having one organization in the lead in implementing the service charter and leading by example.
**3. Chain characteristics**	
*A.*	*Structure of the chain*: The number of organizations in the chain, the degree of competitiveness and the division of power within the chain. Plus, the way the chain has dedicated organizational units for stroke services in the involved organizations.
*B.*	*Extent of integration*: The way processes in the chain are standardized and the chain works as a coordinated integrated care and patient journey.
*C.*	*Characteristics of patients*: The age of patients, their diseases, their physical and emotional condition and the effects of these aspects on the way they want to be involved in the development of the service charter and are interested in using it.
**4. Steering and managing the project**	
A.	*Project structure*: Having a well-structured project structure and committees involving all participating organizations on the strategic, tactical and operational levels.
B.	*Steering Committee*: Having a steering committee including members of all organizations with a broadand uniform mandate. This steers and systematically monitors progress in all phases of development using measures to realize the content of the charter and sustain the change.
*C.*	*Project leader*: Having an independent, stimulating and active project leader in charge of the daily coordination of the project.
*D.*	*Support*: Having experienced support from the project organization that infuses know-how and delivers supporting activities.
**5. The way of development and implementation**	
*A.*	*Action plan*: Having an action plan for all measures on both chain and organization levels.
*B.*	*Timing*: Having the right timing: not so fast that commitment is not obtained, and not so slow that momentum is lost. Ensuring that all organizations work at the same pace.
*C.*	*Improvement actions*: Investing the attention, time and energy to implement all the measures necessary to realize the content of the service charter in all organizations in a uniform manner.
*D.*	*Sustaining*: Regularly and objectively measuring the structural realization of the content of the service charter on the chain and organizational levels.
*E.*	*Continuous improvement*: Regularly evaluating and updating the service charter to match changing demands and preferences of patients. Taking corrective actions if necessary as part of the regular quality control of the chain.
**6. Patient focus**	
*A.*	*Patient-focused service charter*: The content of the service charter is based on needs and preferences of patients. Further, service standards are only promised that can be realized in practice. If standards are not met, there is compensation.
*B.*	*Patient research:* The chain uses methods to research the needs and preferences of patients that give valid and reliable results for the entire patient group.
*C.*	*Patients understand the service charter:* Using effective means to inform patients at the right moments about the philosophy and content of the service charter in order that patients fully understand and can use it.
*D.*	*Continuous feedback from patients:* Using methods to obtain continuous patient feedback on the realization of the service standards.
**7. Employee focus**	
*A.*	*Motivation and stimulation*: Managers motivate and stimulate employees over the service charter.
*B.*	*Commitment of employees*: Creating commitment in each employee to the goals and content of the service charter. Achieved by using the power of opinion leaders, involving all employees, creating a sense of urgency and defining a service charter that employees believe to be realizable.
*C.*	*Willingness to realize the service standards*: There has to be a willingness to ensure the culture change necessary to achieve the patient focus. It is essential that each employee sees the promises in the service charter as his/her personal commitment/promise to each patient every day.

As illustrated in Figure 1, the first three categories focus on the basic foundations for developing and implementing a service charter in an integrated regional stroke service. The fourth category addresses how to manage the project organization, and the final three categories address the way to develop and implement a service charter with a special focus on patients and employees. Of these seven categories, the third category ‘chain characteristics’ has a special position since it contains three properties that describe the context of the chain and which have an impact on the development and implementation but cannot be influenced during it. The seven categories will be briefly described below. These are illustrated by quotes of the participants that best describe the general opinion of the participants.

### Strategy and managerial commitment

A fundamental difference between the development and implementation of a service charter on the chain level with that in a single organization is that not one but several, in this case five, boards of organizations with different cultures and views on quality control, quality improvement and patient-centeredness are involved in the project. Aligning the visions and goals of using a service charter is much more difficult and important in such situations. In our case subject, a well-structured approach was used between 2009 and 2012 with many enthusiastic project members involved and this resulted in the first service charter for a healthcare chain. The data show that a number of aspects concerning strategy and managerial commitment are important. The first is a clear understanding by management and board members of the impact of the concept on the organization. The formal objectives in using the service charter for the integrated stroke service were to improve patient-centeredness and patient satisfaction. In order to realise these two objectives, it is crucial that each participating organization embraces them. As one of the steering committee members stated: ‘*The most important condition is that the organization is really interested in the content of the service charter and wants it to actually improve care, not just so they can say they have one. This is an important difference. Depending on their goals, organizations act differently and this complicates implementation. Depending on their goal, organizations take different actions, facilitate the implementation differently and communicate differently’.* In order to stimulate the development and implementation, it is important to allocate sufficient appropriate resources to it.

### Chain chemistry

A specific category of enablers for developing and implementing a service charter in a chain is the importance of having the right chemistry between the organizations involved. Here, an important enabler is trust, trust in each other and that the implementation will work. As one steering committee member expressed it: *‘If there is no basis for trust, don’t start with it’.* The commitment of all organizations to jointly develop and implement a single service charter based on compromises and consensus is essential. As such, it is necessary to put the interests of the chain above those of the individual organizations. As one of the members of the taskforce put it: *‘It is important that each partner in the chain is willing to give up some of its own advantages’.* Further, openness and transparency are important. Here, the organizations involved were informed about patient satisfaction in the other organizations, teams visited each other’s organization in the chain to learn from their practices and certain procedures were standardized. In achieving this, feelings of competitiveness might have negative consequences. As a steering committee member of one of the specialized nursing homes stated: *‘Our cooperation is now good but the government is promoting competition. One effect is that we, as similar specialized nursing homes, might be forced to compete’.* Finally, participants stated that it is important that one organization takes the lead in the development and implementation.

### Chain characteristics

The characteristics of the chain have an effect on the development and implementation of the service charter. As one of the steering committee members stated: *‘It is much easier to implement a service charter with two organizations than with five of which three undertake the same activities’.* Besides the structure of the chain, the extent of integration is an important enabler. This chain consists of a patient’s journey through several organizations resulting in several logistical interdependencies among the organizations. Having standardized processes makes developing and implementing a service charter much easier. This is illustrated by one of the steering committee members: *‘Still today, our processes are not sufficiently structured. Before implementing the service charter we should have structured and standardized our processes in the chain’.* Another chain characteristic concerns the kind of patients. In this instance, they are mainly frail and elderly with an extreme focus on their health following a stroke. Some respondents expressed doubts about the effectiveness of a service charter for this patient group. Many patients, they felt, would not be interested in the content, would not consult it and would not react if promises were not kept.

### Steering and managing the project

Developing and implementing a service charter within an integrated stroke service involving five organizations demands an extensive project organization. Here, the regional stroke service set up a project structure consisting of a steering committee, a project leader, a taskforce and support on the chain level and a working group within each organization. Participants in our research believed this extensive project organization to be essential for achieving commitment in all organizations. The steering committee has an important role in this structure. To give it sufficient power, it is necessary for each organization to be represented on this committee with broad and uniform mandates. As one of the steering committee members observed: *‘What you see is that there are differences between the organizations. If we have a mandate, we have the authority to use it and take all necessary actions. In other involved organizations, members of the steering committee have a mandate, but it is very limited. The result is that these people have to check decisions with their superiors, and that takes time’.* Further, the project leader has a crucial role. Several respondents stated that it is important to have an independent project leader who is not working for one of the participating organizations and with enough authority to act as a trouble-shooter when necessary.

### The way of development and implementation

Respondents mentioned several enablers relating to the way of developing and implementing a service charter. Having a specific and detailed planning on the chain and organizational levels was often mentioned. Since all the organizations had to commit to the content of the charter, the organizations spent a lot of time discussing the content and ensuring that there was full commitment to the promises in the charter. As a member of the task force explained: *‘We have listened carefully to each other and have given each organization adequate room for comments’*. Further, it is important to use a structured and well-coordinated approach in all phases of the development and implementation. This is required to ensure that the coordination does not stop once the service charter has been defined on the chain level but is extended to making the service charter work and then sustaining it. Participants commented that they underestimated the resources necessary for making the service charter work. A steering committee member commented: ‘*My general criticism is that we spent a lot of time and attention on the development, describing and communicating the service charter and profiling our organizations, but we underestimated the effort required to make the service charter really work. We thought we could simply tell our employees who have to work with the service charter and they would do it. We have learnt that it doesn’t work that way. We have learnt that making a service charter work is difficult: making the service charter work takes as much or even more time and energy than developing it.’*

### Patient focus

Most of the respondents expressed the view that patient needs should be central when developing the service charter. As such, the involved organizations had conducted a survey, organized focus groups and held in-depth interviews prior to implementing the charter. The goals in conducting this extensive research were to gain a sense of current patient satisfaction and to determine the desirable content of a service charter. The development of the charter in this integrated stroke service showed that qualitative research is essential for gaining an in-depth understanding of patients and their preferences. Further, the research needs to be anonymous and easy for the participants. For the frail elderly patients, it was important that the research was anonymous in order to create a safe environment in which they could be open. Furthermore, making it easy for patients to participate by visiting them at home was important. As such, in this instance, focus groups appeared to be less appropriate than individual interviews.

For the service charter to work in daily practice, it is important that patients are informed about it. The method and intensity of communicating the service charter should be the same in all organizations. One of the members of the taskforce who had spent a lot of time and energy on personally informing patients about the service charter explained: *‘It is personally explained to new patients what a service charter is, what our promises are, why we are doing this, what the patient can do when we do not deliver and that we offer small compensation in these cases’.* This was to ensure that patients knew about the service charter and could use it when interacting with employees on the basis that this would lead to concrete improvements. At the time of our research, the chain had yet to implement a well-structured system for evaluating the realization of the service standards for each patient. Participants recognized that this is key for the effective use and sustainability of the charter. A taskforce member observed: *‘We should have a standard evaluation instrument to check the realization of the promises in the service charter with each patient when or after leaving the organization’.*

### Employee focus

Also focusing on employees during the development and implementation is very important since they are the ones who will make the content of the charter a reality in their daily contacts with patients. Managers have a crucial role in creating motivation, enthusiasm and a sense of urgency. As one of the steering committee members stated: ‘*Assemble your employees and convince them of the necessity of the service charter’*. A member of the taskforce provided an illustration of this happening where a manager had gathered his employees together and passionately explained, the background, the reasons for and opportunities of the service charter. This had convinced many employees.

To gain the commitment of employees it is first important to involve them in the working groups. However, this is not enough as the experience of several of the involved organizations is that all employees, both nurses and physicians, have to become involved. Just relying on working groups is insufficient. When forming working groups it is also important to involve not only volunteers but also the opinion leaders of teams since they are able to contribute to changing the culture. The aim of all this is to achieve the full realization of the contents of the service charter in daily practice. The different starting positions of the various organizations and teams within them seem to play a role. There were organizations where the service charter fitted perfectly with their patient-centred culture but others where it seemed not to be fully accepted or understood. Within individual organizations, different teams also accepted the philosophy and content of the charter in various ways.

## Discussion

This exploratory research has resulted in a framework of enablers for the development and implementation of a service charter for an integrated stroke service. It consists of seven categories of enablers containing 27 properties. The results of our research contribute to the literature on service charters, patient-centred integrated care and the implementation of innovations.

Thomassen et al. developed a Public Service Charter Implementation Framework consisting of three main clusters and ten sub-clusters of enablers. This was the first empirical research on enablers for the effective development and implementation of a service charter that focused on individual organizations. The current research has extended the knowledge on this subject by conducting research on the development and implementation of a single service charter covering an integrated care setting. The results show that the enablers are not only related to the organizational level but that there are additional enablers on the chain level related to strategy and managerial commitment, the chemistry in the chain and the characteristics and context of the chain.

Our research has contributed to knowledge on improving patient-centeredness and patient satisfaction in an integrated care setting. Along with functional status, quality of life and costs, patient satisfaction is one of the most important outcome measures of integrated care. Nevertheless, in research on integrated care, patient satisfaction and patient centeredness have received little attention. As Howarth and Haigh [31] observe: ‘evidence of patient centrality from the service user perspective has not been satisfactorily explored’. Only a few studies have researched the effects of implementing the concept of integrated care on patient satisfaction. Rosendal et al. [32] concluded from their study that patient satisfaction increased after implementing integrated stroke care. The further optimization of integrated stroke care, in order to enhance patient satisfaction, is seen as an important field for research worldwide [33]. The use of service charters could be seen as a concept for further optimizing patient centeredness in integrated care. It is a means for improving communication with patients and the patient-centeredness of employees and processess.

The effectiveness of an implementation depends on power, culture and structure [34]. Power reflects the capacity to influence people in a desired direction. In the stroke service investigated, nobody is the ‘boss’. None of the organizations has enough power to force the others to participate fully. A consequence is that the content of the service charter is based on consensus, and the speed of development and implementation is determined by the slowest organization. Power in the chain would seem to be helpful in achieving a successful implementation.

Culture reflects the set of values, guiding beliefs, understandings and ways of thinking shared by members of the chain. The main goals of using the service charter were to increase patient-centeredness and patient satisfaction. Developing and implementing this concept and achieving its goals seemed to be smoother in those organizations that already had a dominant patient-centred culture because the charter corresponded with existing values, strategies, goals, skills and ways of working. Culture will also be reflected in the actual organizational goals of using a service charter. The alignment of goals within the chain is a core factor for effective change, a misalignment is a source of potential tension. Congruent goals among all five involved organizations are essential for an effective implementation. Although the formal goals were to increase patient-centeredness and patient satisfaction, some of the organizations had other goals such as having a service charter as a marketing tool or avoiding becoming separated from the chain through non-participation. This resulted in differences in the actual implementation of the necessary measures and in the effectiveness of using the service charter in the various organizations. Having a patient-centred culture and alignment on objectives seems to be very important when using a common service charter.

Finally, structure also has an impact on development and implementation. Implementing a service charter across five organizations forces micro-level standardization of practices within the healthcare system. Leutz [35] has described three prototypical models and phases in increasing integration: linkage, co-ordination and full integration. Based on the results of our interviews, we were able to conclude that the stroke service acts somewhere between linkage and co-ordination phases. The consistent use of a service charter is probably best achieved when embedded in a chain in the co-ordination or full integration phases. In our case, the development of the service charter was a joint operation, but its implementation was carried out independently by each organization. Due to differences in the approaches adopted, this led to some tensions in the chain. As such, it is important that there is a central approach not only in the development of the service charter but also in the implementation of the measures in order to realize and sustain the charter’s content in all the involved organizations.

Our research shows that beside power, culture and structure also mutual trust influences the effectiveness of an implementation. Trust among organizations is essential for the development and implementation of a joint service charter. Managing the integration necessary for the implementation of the service charter has been shown to be more of a process of deliberation and negotiation between organizations than one of ideology and prescription [36]. A lack of trust in this process could be a barrier and could block the necessary integration [37]. Trust can be developed by working together and jointly achieving successes. However, miscommunications as a result of prioritizing one’s own organization, a lack of communication and uncertainty can all be barriers to integration and sources of distrust [38].

Five organizations delivering health care in two of the three phases of an integrated regional stroke service have developed and implemented a service charter. Our research is based on the opinions of experienced practitioners working in these five organizations As such, the external validity of the study is limited: the framework could be improved by conducting similar studies on integrated healthcare and on chains in general. Since the goal of our research was to determine the organizational enablers, we have included experienced managers and employees. In our research we did not use the opinions of patients and their relatives. Follow up research could study the expectations and preferences of this important group on the for the patient relevant aspects of the implementation. By using qualitative research methods preferences concerning e.g. how to communicate and use a service charter could be researched. Furthermore research could be done on the actual effectiveness of service charters. Do they really contribute in improving the patient-centeredness and increasing patient satisfaction? This follow-up research could address the relationships between the enablers and actual results in terms of patient-centeredness and patient satisfaction. In a healthcare setting no research on this issue has been done till now.

## Conclusions

This first exploratory study into the enablers of the effective development and implementation of a service charter for a chain has identified seven categories of enablers made up of 27 properties. This was based on a single case study in the Netherlands. This was the only integrated care chain using a single service charter that we could find. Nevertheless, the results of our explorative study provide an initial framework for the development and implementation of service charters in integrated care settings. This research contributes to the literature on service charters, on patient-centeredness in integrated care and on the implementation of innovations.

## Competing interests

The authors declare that they have no competing interests. There has been no funding from commercial organizations for this research. None of the researchers occupy roles in any of the five organisations that take part in this research.

## Authors’ contributions

JPT contributed to the conception and design of the study, data analysis, interpretation of the data and in drafting the paper. KA and SW contributed to the conception, the design of the study and in drafting the paper. Finally, all authors read and approved the submitted manuscript.

## Pre-publication history

The pre-publication history for this paper can be accessed here:

http://www.biomedcentral.com/1472-6963/14/141/prepub
